# Supplementary material for: Relative assessment of cloth mask protection against ballistic droplets: A frugal approach
Source: PLoS One. 2022 Oct 4;17(10):e0275376. doi: 10.1371/journal.pone.0275376 (PMC9531801; doi:10.1371/journal.pone.0275376)
Supplement: S1 File — Section A Visualization of the free jet. Section B Technical details. Section C Uncertainty analysis. (PDF) [file pone.0275376.s001.pdf]

S1 Supporting Information. Experimental details.

A. Visualization of the free jet

The jet was visualized by illuminating with a light “cone” produced by a green laser (λ = 532 nm and peak power of 50 mW). The images were acquired at 30 fps using a NIKON D5100 camera. Fig A illustrates the evolution of the jet based in its visualization.

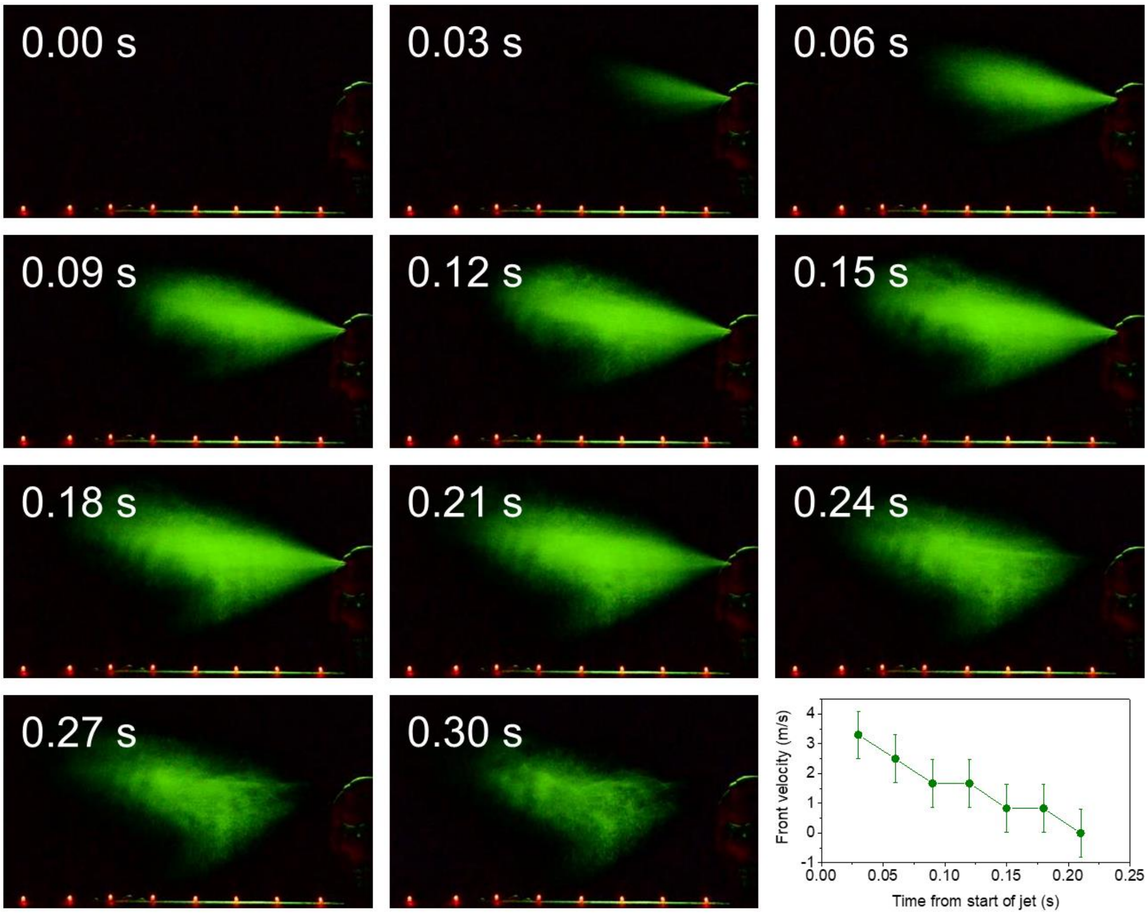

Fig A. Visualization of the free jet. The 11 photographs are a time sequence of snapshots of the free jet, visualized using laser light. The time count starts when the nozzle starts generating liquid. In each of the snapshots, the green light indicates the presence of liquid particles emitted by the nozzle (located at far right within each image). The red spots are LEDs located at a distance of 5 cm from each other. Notice that the spray can be assumed as a cone making an angle of 10° around the horizontal direction. Bottom right panel: velocity of the jet front as time goes by.

B. Technical details

Construction and procedure details

The bucket used was 17 cm long, with a circular base of 15 cm diameter, and a circular aperture of 17 cm diameter at the opposite end. A circular hole of 2.5 cm diameter was made at the center of the base using a punch tool, so the liquid jet could pass through (other shapes would have worked as well).

The blocking materials were set in place by using rings cut off from another, identical bucket. The textiles were gently presented against the aperture, and then stretched by pushing the ring into place.

The spray bottle contained blue-colored water obtained by dissolving 0.5 g of methylene blue powder in 1 L of tap water. Before each experiment, a 75 g/m^2-density white paper sheet containing a marked area of 5×5 cm^2 was placed on the device (see Fig 1B), ensuring that the center of the area was aligned with the bottle nozzle. As the nozzle was shot, droplets hit the paper sheet, leaving blue stains on it.

The 5×5 cm^2 area (referred to as “screen”), containing the stain patterns, were scanned with a resolution of 3200 dpi and 24-bit RGB color, using a scanner EPSON model Perfection V370 Photo. Then, the image was negated, and its red component binarized. As the stains are blue, this component provided the best contrast between the stains and the background. After careful comparison between the original patterns and the binarized images, we chose a binarization threshold of 0.3. See [47] for accessing the code used in the image processing along with a detailed documentation.

If the image with the stain pattern was subtracted from the scan of that very same sheet of paper made before the droplets were shot (in order to account for intrinsic lack of homogeneity of the paper), the results for BBC were negligibly different.

Micrographs of blocking materials

Micrographs of different blocking materials, shown on the left column in Fig 2, were obtained using a Dino-lite Premier digital microscope.

C. Uncertainty analysis

In the calculation of $\langle\overline{p_{no}}\rangle$ and $\langle\overline{p_o}\rangle$ (both referred as $\langle\bar{p}\rangle$ from now on, as they are computed in the same way), there are two sources of Type B standard uncertainty: the stained paper scan resolution and the finite precision with which the program reads the pixel values.

In the case of the former, a test was made to check if an increase in the image resolution would affect significantly the values of $\langle\bar{p}\rangle$ calculated in the experiments. In order to do so, two raster images were created from the same vector using resolutions of 3200 and 6400 dpi (see Fig C) and $\langle\bar{p}\rangle$ was calculated on both of them. The difference between both was around 0.1% of the value of the 3200 dpi image, so the influence of this source of uncertainty can be neglected as compared to the influence of the fluctuations between repetitions of the same experiment, as we will see below.

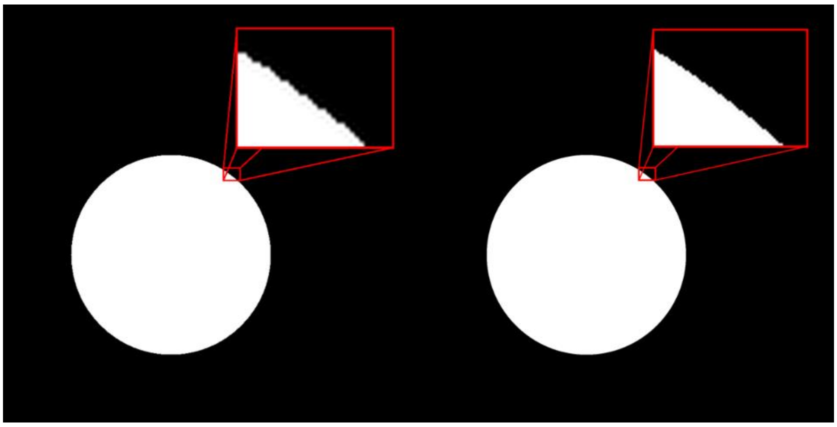

Fig C. Uncertainties in image digitizing. Two raster images created from the same white disk on a black background vector graphics, simulating the stains in the binarized images of the experiments. Left panel: 3200 dpi resolution image. Right panel: 6400 dpi resolution image. In the insets it can be seen the difference between the analog regions in the disks boundaries.

The program used for image processing reads by default the pixel values with a precision of 15 significant decimal digits. As the pixel values are within the interval [0,1] the difference between two pixels whose values differ up to $R = 10^{-15}$ can be detected. Due to binarization, this resolution would only affect the pixels whose values are within the interval $[\mathrm{Th} - \mathrm{R}, \mathrm{Th} + \mathrm{R}]$ being Th the binarization threshold used in the image processing. In a typical image, the proportion of pixels that satisfies this condition varies from zero to 10^{-15}, so the influence of that source of uncertainty can be also neglected. In summary, the influence of Type B is negligible compared to the Type A standard uncertainty on the evaluation of $\langle\overline{\mathrm{p}}\rangle$'s combined standard uncertainty.

For all experimental configurations, $\langle\overline{\mathrm{p}}\rangle$'s Type A standard uncertainty was computed using the well-known positive square-root of the unbiased estimator for the variance of a sample divided by its length. Each experiment was repeated 3 times and a 95% confidence interval was selected for the calculations, so according to Eq. (1), the formula used for the expanded uncertainty of BBC is

$$\mathrm{u(BBC)} = \left[\mathrm{t}_2 \left(\frac{1}{\langle\overline{\mathrm{p}_{\mathrm{no}}}\rangle^2} \mathrm{u_A}^2(\langle\overline{\mathrm{p}_{\mathrm{o}}}\rangle) + \frac{\langle\overline{\mathrm{p}_{\mathrm{o}}}\rangle^2}{\langle\overline{\mathrm{p}_{\mathrm{no}}}\rangle^4} \mathrm{u_A}^2(\langle\overline{\mathrm{p}_{\mathrm{no}}}\rangle) \right)^{1/2} \right] \times 100\% \qquad \mathrm{(C1)}$$

where $\mathrm{t}_2 = 4.30$ is the factor corresponding to the confidence interval of choice and the effective degree of freedom.
